# Intermittent Theta Burst Stimulation to the Primary Motor Cortex Reduces Cortical Inhibition: A TMS-EEG Study

**DOI:** 10.3390/brainsci11091114

**Published:** 2021-08-24

**Authors:** Zhongfei Bai, Jiaqi Zhang, Kenneth N. K. Fong

**Affiliations:** 1 Department of Rehabilitation Sciences, The Hong Kong Polytechnic University, Kowloon, Hong Kong; zhong-fei.bai@connect.polyu.hk (Z.B.); jack-jq.zhang@connect.polyu.hk (J.Z.); 2 Department of Rehabilitation, Shanghai Yangzhi Rehabilitation Hospital (Shanghai Sunshine Rehabilitation Center), Tongji University, Shanghai 201619, China

**Keywords:** transcranial magnetic stimulation, TMS-evoked potentials, primary motor cortex, cortical inhibition

## Abstract

Introduction: The aim of this study was to reveal the effects of intermittent theta burst stimulation (iTBS) in modulating cortical networks using transcranial magnetic stimulation and electroencephalography (TMS-EEG) recording. Methods: Eighteen young adults participated in our study and received iTBS to the primary motor cortex (M1), supplementary motor area, and the primary visual cortex in three separate sessions. A finger tapping task and ipsilateral single-pulse TMS-EEG recording for the M1 were administrated before and after iTBS in each session. The effects of iTBS in motor performance and TMS-evoked potentials (TEPs) were investigated. Results: The results showed that iTBS to the M1, but not supplementary motor area or the primary visual cortex, significantly reduced the N100 amplitude of M1 TEPs in bilateral hemispheres (*p* = 0.019), with a more prominent effect in the contralateral hemisphere than in the stimulated hemisphere. Moreover, only iTBS to the M1 decreased global mean field power (corrected ps < 0.05), interhemispheric signal propagation (*t* = 2.53, *p* = 0.030), and TMS-induced early α-band synchronization (*p* = 0.020). Conclusion: Our study confirmed the local and remote after-effects of iTBS in reducing cortical inhibition in the M1. TMS-induced oscillations after iTBS for changed cortical excitability in patients with various neurological and psychiatric conditions are worth further exploration.

## 1. Introduction

Transcranial magnetic stimulation (TMS) is a pain-free and non-invasive brain stimulation technique [1]. Theta burst stimulation protocols are forms of patterned repetitive TMS, of which intermittent theta burst stimulation (iTBS) was found to produce a rapid and powerful after-effect in facilitating the cortex outlasting the stimulation period, whereas continuous theta burst stimulation (cTBS) shows a suppressive effect [2]. Accordingly, mechanisms underpinning the modulatory effect of iTBS and cTBS have been investigated in previous studies. Huang et al. [3] proposed that iTBS and cTBS produce different patterns of calcium influx to postsynaptic neurons through NMDA channels, resulting in long-term potentiation and long-term depression, respectively [3]. Furthermore, it was found that the modulatory effect of iTBS increased significantly when Gamma-aminobutyric acid (GABA) receptors were blocked [4], revealing the essential role of GABA inhibitory circuits in the aftereffect of iTBS.

Motor-evoked potentials (MEPs) are recorded in contralateral hand muscles when suprathreshold TMS pulses are applied to the primary motor cortex (M1). The amplitude of MEPs can be used to quantify the excitability of corticospinal tracts [5]. For instance, an increment of MEP amplitude can be observed after iTBS to the M1 [2]. MEPs are also used to probe the connectivity between the M1 and other regions. For instance, MEP amplitude was enhanced after high-frequency repetitive TMS to the supplementary motor area (SMA) [6], while low-frequency repetitive TMS to the SMA had the opposite effect [7]. In addition, paired-pulse TMS measures, employing a test pulse following a conditioning pulse, for example, short-/long-interval intracortical inhibition (SICI/LICI), intracortical facilitation, and interhemispheric inhibition, are used to investigate the intracortical facilitation and intracortical/interhemispheric inhibition of the M1 [1]. However, these TMS measures based on MEPs highly rely on corticospinal outputs, restricting to probe brain regions beyond M1. In addition to the huge variability of these measures [8], there is another limitation that MEP-based measurements cannot yield a pure cortical response without the contamination of spinal excitability [9,10].

Concurrent TMS and electroencephalography (TMS-EEG) recording is a new approach to directly probe the activity of cortices [9]. It was confirmed that TMS pulses with even subthreshold intensities applied to the M1 can consistently evoke a series of time- and phase-locked positive or negative deflections, including N15, P30, N45, P55, N100, and P180, termed TMS-evoked potentials (TEPs; [11]). Although the mechanism underlying these peaks has not been clearly understood, pharmacological studies have provided insights into the relationship between TEP peaks and cortical excitability, summarized by Darmani et al. [12]. It was found that positive modulators and antagonists of GABAA receptors increased and decreased the amplitude of N45, respectively [13,14], while baclofen, a GABAB receptor agonist, increased the N100 amplitude significantly [13]. These studies indicated a strong relationship between later TEP peaks (N45 and N100) and intracortical inhibition mediated by GABA receptors, which may be an underlying mechanism of the late TEP peaks. Most recently, a study by Hui et al. showed that baclofen could decrease TMS-induced interhemispheric signal propagation (ISP), probably a potential neurophysiological biomarker reflecting interhemispheric inhibition mediated by GABAB receptors [15].

Previously, many studies explored the modulatory effect of TMS using TEPs. An early study found that high-frequency repetitive TMS increased the amplitude of MEPs accompanied by increased global mean field power (GMFP) of TEPs before 50 ms [16]. In contrast, low-frequency repetitive TMS appeared to increase the amplitudes of P60 and N100 when delivered to the M1 compared with the primary visual cortex (V1; [17]). Regarding the effect of iTBS, Chung et al. found that only iTBS with an intensity of 75% of resting motor threshold (RMT) to the dorsolateral prefrontal cortex could significantly increase the N100 amplitude [18]. In addition, the effect of iTBS to the cerebellum can be sensitively detected by an increased N100 amplitude [19]. However, the first study investigating iTBS to the M1 using TMS-EEG did not find any significant changes on TEPs in a group of older healthy volunteers [20].

At present, iTBS is increasingly popular in brain research and clinical application. A recent meta-analysis confirmed that iTBS could enhance cortical excitability of the M1 in healthy people; however, the effects on intracortical facilitation and SICI were not consistently reported [21]. In view of the limitations of MEP-based measures, TMS-EEG may be more informative to probe intracortical excitability than MEP-based measurements. In the current study, TMS-EEG was carried out pre- and post-iTBS to three brain regions—the M1, SMA, and V1 (i.e., control condition). This study aimed to investigate the effect of iTBS on the M1 and SMA in modulating cortical facilitation and cortical inhibition measured by TEPs and TMS-induced oscillations, as well as motor performance in young, healthy individuals.

## 2. Methods

### 2.1. Participants

Eighteen young adults (12 males) with normal or corrected-to-normal vision, aged between 20 and 35 years, were recruited by convenience sampling. All of them were non-smokers, right-handed according to the Edinburgh Handedness Inventory [22], and had no medical history of neurological or psychiatric illnesses. We excluded participants who were taking neuropsychiatric drugs and those who had contraindication to TMS [23]. Written informed consent was obtained from all participants and this study was approved by the Human Research Ethics Committee of the Hong Kong Polytechnic University (Reference Number: HSEARS20190812001).

### 2.2. Procedures

**Figure 1:**
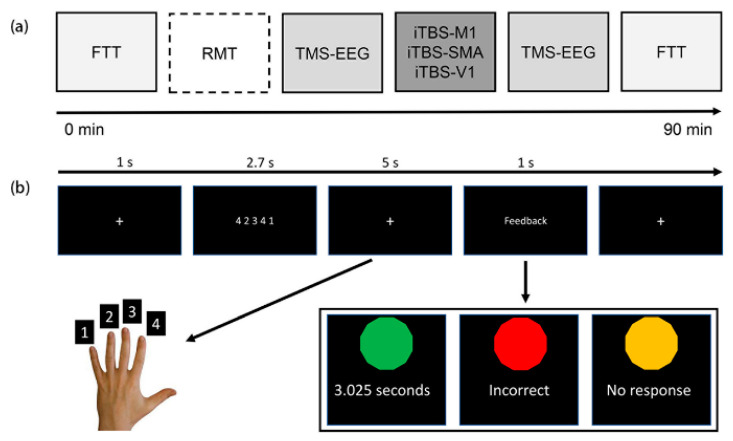
Experimental procedures and the finger tapping task. (**a**) TMS-EEG recording of 90 single-pulse of SMA and V1. Resting motor threshold was determined in the first session only and the stimulation intensity was maintained the same for the conditions that followed. (**b**) Illustration of the finger tapping task (only the left hand is shown). FTT: finger tapping task; RMT: resting motor threshold; TMS-EEG: transcranial magnetic stimulation and electroencephalography; iTBS: intermittent theta burst stimulation; M1: primary motor cortex; SMA: supplementary motor area; V1: primary visual cortex.

All participants attended three sessions with intervals of at least five days or longer. The order of iTBS to the M1, SMA, and V1 was pseudorandomized across participants. The V1 condition was set for controlled comparison as previous studies showed that neither iTBS nor low-frequency repetitive TMS to this area could modulate cortical excitability of the M1 [17,24]. The experimental procedure is depicted in Figure 1a. As the non-dominated hand is relatively less dextrous than the dominated hand and the non-dominated hemisphere has lower cortical excitability than the dominated hemisphere [25], the non-dominated hemisphere may benefit from iTBS more easily. In addition, the non-dominated hand could also mimic the impaired motor function after unilateral stroke. Therefore, the right M1 was selected to be the target for iTBS. EEG was concurrently recorded during 90 TMS biphasic pulses delivered to the right M1 pre and around 7–10 min post-iTBS. In addition, a finger tapping task (FTT) aiming to measure the effect of iTBS in motor performance was conducted pre- and post-iTBS.

### 2.3. Finger Tapping Task

The FTT test was similar to that in Solopchuk et al. [26] and Kim et al. [27]. Participants were comfortably seated approximately 70 cm away from a monitor. The E-Prime software (version 2; Psychology Software Tools, Inc., Sharpsburg, PA, USA) was used for trial presentation. In each trial, as depicted in Figure 1b, first, the monitor showed a white cross for 1 s, followed by a five-number sequence for 2.7 s. Thereafter, participants were required to input the sequence in 5 s and feedback was given after inputting five numbers. In total, both hands needed to complete 60 trials. Only number combinations in which the same numbers were repeated twice were chosen, and we excluded those having two continuous same numbers. Outcomes of the FTT test were indexed by reaction time (RT) and accuracy (ACC). In addition, normalized improvement was obtained by dividing the Δvalue (i.e., difference, post−pre) by the performance pre-iTBS.

### 2.4. Transcranial Magnetic Stimulation

TMS was carried out using a stimulator (MagVenture A/S, Farum, Denmark) with a 75 mm figure-of-eight coil (C-B60) for RMT measurement, TMS-EEG recording, and iTBS. The coil positioning and orientation on the scalp were consistently monitored by a frameless stereotactic neuronavigation system (Localite TMS Navigator) coupled with a Polaris Vicra infrared camera (NDI, Waterloo, ON, Canada) using the ICBM152 template. As illustrated in Figure 1, RMT of the left first dorsal interosseous muscle was determined in the first session. The coil positioning to the M1 was the hotspot where the most consistent and largest peak-to-peak MEP amplitude was elicited by suprathreshold biphasic pulses. MEPs were recorded using surface electrodes positioned in a belly-tendon montage. RMT was defined as the minimum output intensity, eliciting at least three out of six MEPs higher than 50 µV [28]. The coil was always placed at about a 45° angle away from the midline with the handle pointed backwards and laterally. This orientation can produce a current perpendicular to the axis of the M1 and is optimal to stimulate it [29].

Regarding TMS-EEG recoding procedures, 90 biphasic pulses were delivered with an average interval of 5 s (110% jitter) to the hotspot of the right M1. The stimulation intensity was set at 110% of RMT with reference to previous studies [9,19]. The intensity of iTBS (600 pulses) was set at 70% of RMT as previous studies showed that this intensity was adequate to modulate corticospinal excitability of the M1 [24]. The coil positioning and orientation for iTBS targeting the M1 was at the hotspot. Previous studies determined the site for SMA stimulation was at around 3–4 cm anterior from the vertex (i.e., Cz; [6,30]). In the current study, we localized the right SMA at MNI coordinates of X = 5, Y = −7, and Z = 58 using the navigational system [31]. To effectively stimulate the SMA, the coil was held tangentially to the skull with the handle predominantly pointing to the left [6,31]. The site for V1 stimulation was around 3 cm anterior from the inion [17], and the coil was held tangentially to the skull with the handle predominantly pointing to the right.

### 2.5. TMS-EEG Recording

A TMS-compatible EEG system (eego™, ANT Neuro, Hengelo, The Netherlands) with 63 Ag/AgCl electrodes mounted according to the international 10–20 system was used to record the EEG response following TMS pulses. The impedance of all electrodes was maintained below 10 kΩ throughout the experiment. The EEG data were referenced online to CPz and grounded to AFz, digitized at a sampling rate of 8 kHz. All participants wore earplugs and white noise was played in the earplugs to reduce the contamination of both external noise and auditory-evoked potentials produced by TMS clicking noise, which may mask the N100 peak of M1 TEPs [32]. Moreover, a thin piece of foam was placed under the coil to minimize TMS-decay artifacts by avoiding direct contact with electrodes [9]. To avoid eye movements, participants were required to gaze at a black cross with a white background almost 2 m away.

### 2.6. Data Processing

The EEG data were processed offline using EEGLAB [33], TESA extension [34], and FieldTrip [35] in Matlab 2016a (The MathWorks, Portola Valley, CA, USA) following the steps proposed by Rogasch et al. [34] that are capable of removing artifacts including the TMS-decay artifacts and others. M1 and M2 electrodes were removed from the raw data and the data of the remaining 61 electrodes were used for analysis and plotting. In addition, electrodes with contamination throughout the TMS-EEG recording period were removed and interpolated using the spherical method. The continuous EEG data were segmented around TMS pulses (−1000 to 999 ms), and baseline corrected (−500 to −100 ms). Then, the data around TMS pulses (−2 to 15 ms) were removed and interpolated using the cubic method. All trials were visually inspected and the trial with large artifacts (muscle, electrical, electrode movement) was removed. The mean numbers of trials in the M1, SMA, and V1 conditions were 89.6 (SD = 0.7), 88.2 (SD = 4.1), and 89.3 (SD = 1.1), respectively. Thereafter, two rounds of independent component analysis (FastICA, systematic approach, and tanh contrast function) were carried out. As the raw EEG signals were mixed with the TMS-decay artifacts with amplitudes larger than neural activity in several orders of magnitude, a low-pass filter could produce a small ringing noise and the capability of independent component analysis in decomposing neural components could also be weakened. Therefore, the purpose of the first round was to remove the largest TMS-decay artifact detected by a semi-automated component classification algorithm implemented in TESA [34]. Then, the EEG data were bandpass filtered (1–80 Hz) and bandstop filtered (48–52 Hz) using a second-order Butterworth filter. FastICA was conducted again to remove remaining artifacts (eye movement, persistent muscle activity, electrode noise) by visual inspection and the semi-automated component classification algorithm. Finally, the EEG data were referenced to common average and TEPs of each participant in each condition were obtained by averaging across trials. With reference to previous literature [9], five time windows were chosen to define the peaks of M1 TEPs: P30 (27–33 ms), N45 (37–43 ms), P55 (48–58 ms), N100 (90–130 ms), and P180 (160–200 ms). To explore the global brain activity following TMS pulses, GMFP of TEPs was computed using the following formula [36]:

$$\mathrm{GMFP}\left(t\right)=\sqrt{\frac{\left[\sum_{i}^{k}{\left(V_{i}\left(t\right)-V_{mean}\left(t\right)\right)}^{2}\right]}{K}}$$

where *t* is time, *V* is the voltage at channel *i*, and *K* is the number of channels.

**Figure 2:**
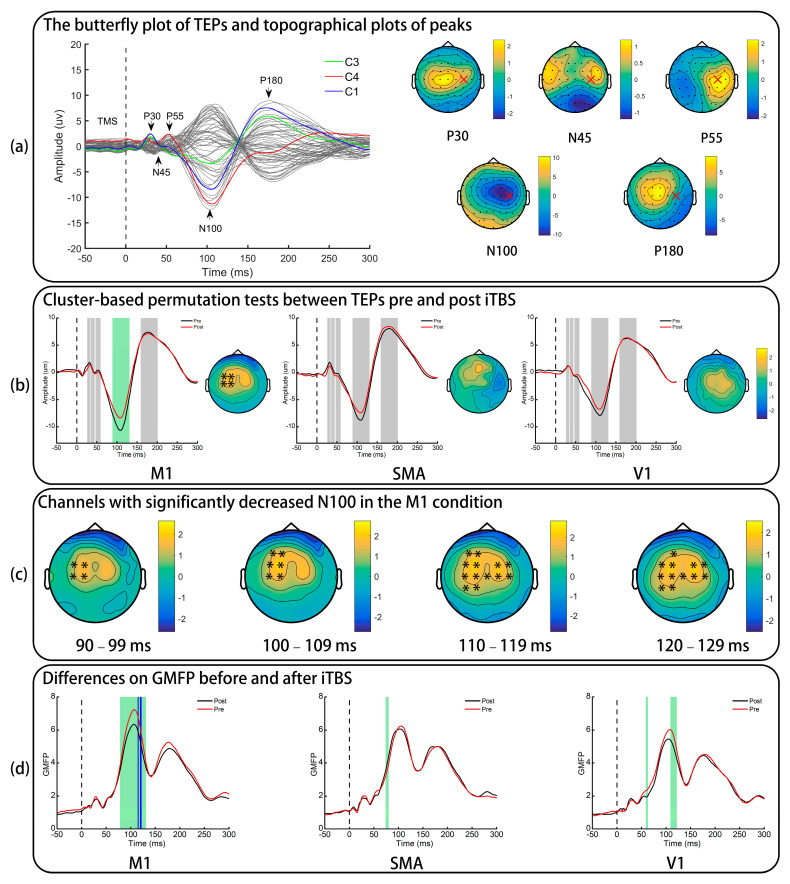
The effect of iTBS in TMS-evoked potentials. (**a**) Only pre-iTBS data of the M1 condition are presented. “x” indicates the stimulation site. (**b**) TEP difference of a representative electrode (FC1). The grey shadows indicate the predefined time windows of five peaks and the green shadow indicates the significant difference in the N100 of M1 TEPs. Topographical plots are the difference of N100 amplitudes (post−pre) and “*” represents electrodes with a significant difference. (**c**) “*” represents electrodes with a significant difference induced by iTBS to the M1 in four small time windows of N100. (**d**) The global mean field power pre- and post-iTBS. Green shadows indicate a significant difference identified by paired *t*-tests and the blue shadows indicate comparisons survived after FDR corrections were applied. TEPs: TMS-evoked potentials; iTBS: intermittent theta burst stimulation; M1: primary motor cortex; SMA: supplementary motor area; V1: primary visual cortex; GMFP: global mean field power.

ISP was calculated by dividing the area under TEP curves in the left hemisphere by that in the right hemisphere, as it was shown to be reliable in recent studies [15,37]. Theoretically, an ISP value below 100% was expected. Given the finding that N100 of M1 TEPs was more robust in the right-lateral area (Figure 2a), we chose C3 and C4 channels as the representatives of the left and right M1s, respectively. Previous studies found that around 10 s was taken for TMS-induced interhemispheric signal propagation [38]. Therefore, the time window between 90 and 120 ms was chosen for the C4 electrode and 100–130 ms for the C3 electrode to account for the delayed interhemispheric propagation.

To obtain TMS-induced oscillations, TEPs were subtracted from all trials [39]. Then, the Morlet wavelet time-frequency method (3.5 cycles, frequency steps of 1 Hz from 5 to 48 Hz, time resolution of 2 ms) was used to convert the temporal signal to TMS-induced oscillatory power. The calculated power of each frequency was normalized by dividing all time bins by the mean of baseline power (−650 to −350 ms; [18]), and an absolute baseline correction was carried out afterwards. After these procedures, the ratio of changes from baseline was gained and stored in each time bin. A normalized power value above 0 indicated synchronized oscillatory power compared with corresponding baselines, and vice versa [40]. Normalized power values of θ (5–7 Hz), α (8–12 Hz), and β (13–30 Hz) bands were calculated by averaging across frequencies [18,39]. As reported previously, GABA drugs could modulate TMS-induced oscillations in α- and β-band in an early time window (30–200 ms) and a late time window (200–400 ms; [39]). Therefore, we also defined these two windows for subsequent analyses.

### 2.7. Statistical Analysis

Statistical analyses were performed using SPSS22 (IBM, Armonk, NY, USA) and FieldTrip in Matlab 2016a [35]. Both one-sample Kolmogorov−Smirnov tests and histogram plots were applied to check for the normality of variables prior to parametric tests. One-way repeated measures analysis of variance (rmANOVA) tests were performed to confirm the comparability of ACC and RT pre-iTBS. To explore the effects of iTBS in motor performance, ACC and RT were subjected to two-way rmANOVA tests, with the main effect of time and the interaction effect of “time × condition”; in addition, one-way rmANOVA tests were also computed using the data of normalized improvement. Post-hoc comparisons between conditions were performed using paired t-tests with Bonferroni corrections (corrected alpha threshold = 0.05/3) if one-way rmANOVA tests were significant.

To assess iTBS-induced modulation in TEPs, non-parametric cluster-based permutation tests by means of the Monte Carlo method were conducted to address the multiple comparisons problem in spatial and temporal dimensions [41]. The alpha threshold to determine whether an electrode-time sample could be clustered was set at 0.05 and at least two neighborhood electrodes with statistical significance were required to define a cluster. Two thousand random permutations occurred and t-values within every cluster were summed up for cluster-level statistics in each permutation. The proportion of random permutations resulting in larger test statistics than the observed one was the significance probability, also known as the *p*-value of clusters. Any positive or negative clusters with *p*-values < 0.025 were considered to show significant difference between TEPs. GMFPs pre- and post-iTBS in each condition were compared by paired t-tests and FDR correction was applied to control the inflation of type I errors caused by multiple comparisons. The statistical analysis of ISP was the same as that of FTT results. Because iTBS to the M1 was found to decrease ISP, which was not expected, we compared the decrement of areas under TEP curves of C3 and C4 using an independent t-test. Non-parametric cluster-based permutation tests with the same parameters for TEPs were carried out again for the statistics in oscillatory power. We also explored the relationship between TMS-induced oscillations and the N100 amplitude of M1 TEPs by Spearman’s rank correlation using pre-iTBS data of the three conditions (*n* = 42).

## 3. Results

Eighteen participants completed all experimental procedures and no adverse events resulting from TMS were noted. The results of the FFT test were analyzed based on all participants. However, the TMS-EEG data of four participants were excluded owing to low signal-to-noise ratio or refractory TMS artifacts in some channels that mixed with neural components and could not be separated by the independent component analysis.

### 3.1. Finger Tapping Task

**Figure 3:**
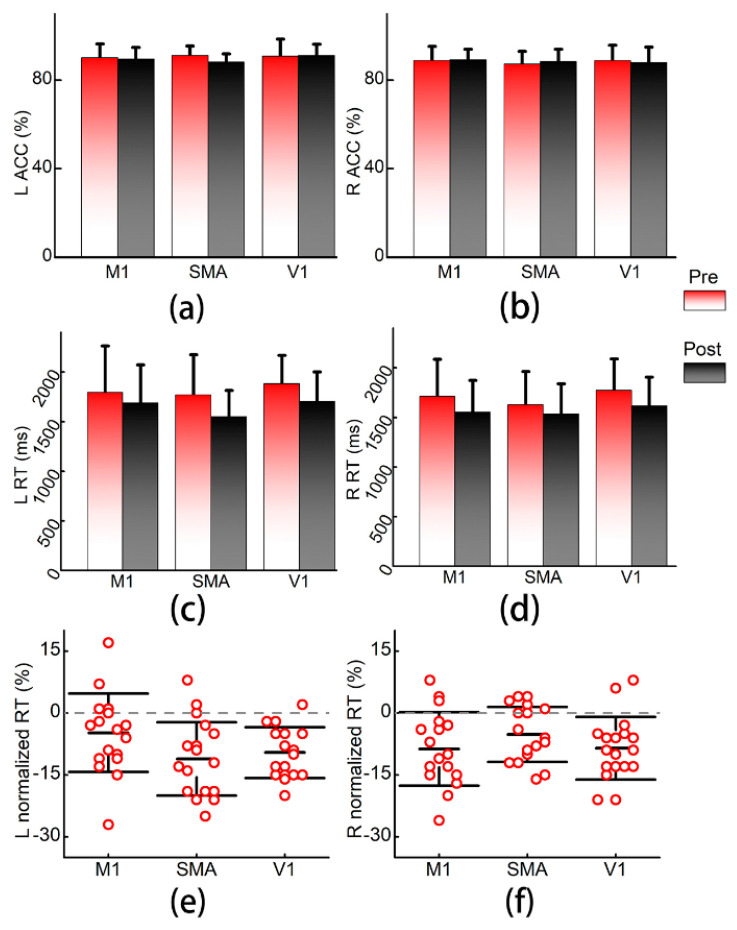
Results of the finger tapping task. (**a**,**b**) Accuracy rates of the left and right hands; (**c**,**d**) reaction time of left and right hands in milliseconds. (**e**,**f**) Comparisons of normalized reaction time. Error bars are standard diversions. L: left; R: right; ACC: accuracy rate; RT: reaction time; M1: primary motor cortex; SMA: supplementary motor area; V1: primary visual cortex.

The results of the FTT test are presented in Figure 3. One-way rmANOVA tests showed that participants had comparable performance on ACC and RT pre-iTBS among the three conditions (all ps > 0.05). Two-way rmANOVA tests did not show significant time (L: F = 2.08, *p* = 0.155; R: F = 0.05, *p* = 0.821) or time × condition interaction (L: F = 1.56, *p* = 0.221; R: F = 0.71, *p* = 0.496) effects with regard to ACC of both hands. Furthermore, normalized ACC also did not significantly differ among conditions (L: F = 2.05, *p* = 0.145; R: F = 0.71, *p* = 0.499).

As expected, significant time effects on RT (L: F = 57.56, *p* < 0.001; R: F = 45.21, *p* < 0.001) were found in both hands, while time × condition interaction effects (L: F = 2.19, *p* = 0.122; R: F = 1.23, *p* = 0.300) were nonsignificant. The normalized RT was not significantly different among conditions (F = 1.33, *p* = 0.278) in the right hand, but a significant between-condition difference in the left hand (F = 3.377, *p* = 0.046) was found. Paired t-tests on normalized RT in the left hand indicated that there were no significant comparison pairs that survived after Bonferroni corrections, but the SMA stimulation tended to result in the most prominent improvement in the left hand (M1 vs. SMA, *t* = 2.57, *p* = 0.020; M1 vs. V1, *t* = 1.98, *p* = 0.064; SMA vs. V1, *t* = 0.56, *p* = 0.581).

### 3.2. TMS-Evoked Potentials

The grand-averaged TEP waveform is presented in Figure 2a and shows five clear peaks at approximately 30 ms (P30), 43 ms (N45), 53 ms (P55), 105 ms (N100), and 180 ms (P180).

The results of cluster-based permutation tests between TEPs pre- and post-iTBS are presented in Figure 2b. It was found that iTBS to the SMA and V1 did not significantly alter TEPs (all ps > 0.025). Although no significant clusters were found in P30, N45, P55, and P180 (all ps > 0.025), iTBS to the M1 resulted in a significant decrease in the N100 amplitudes of M1 TEPs in four channels (FC1, FC3, C1, and C3) in the contralateral hemisphere throughout the predefined time window of N100 (*p* of the cluster = 0.019). The time window of N100 was then segmented into four small windows, 90–99 ms, 100–109 ms, 110–119 ms, and 120–129 ms (Figure 2c), and a significant difference in the ipsilateral hemisphere (FC2, FC4, C2, and C4) was also noted in late phases of N100 (110–119 ms, 120–129 ms; *p* = 0.019).

### 3.3. Global Mean Field Power

GMFP of TEPs is presented in Figure 2d. Point-to-point paired t-tests on GMFP indicated significant difference in all conditions (green shadow). However, only iTBS to the M1 significantly decreased GMFP in the time window of N100 (blue shadow: 115 ms, 120–121 ms) after FDR corrections were applied.

### 3.4. Interhemispheric Signal Propagation

**Figure 4:**
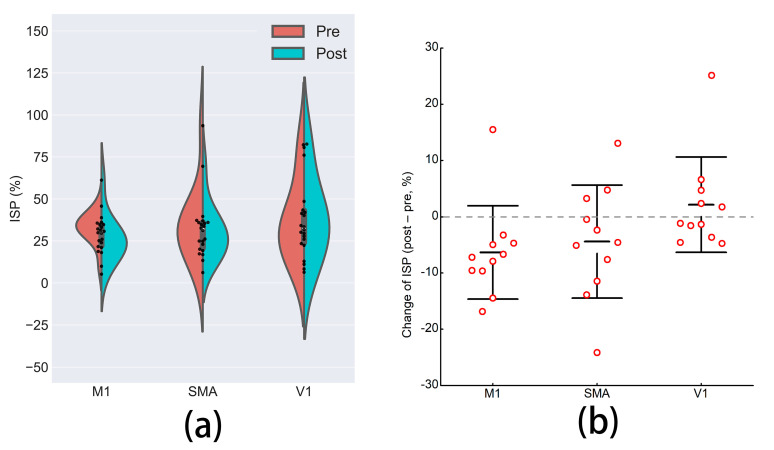
The effects on TMS-induced interhemispheric signal propagation. (**a**) The difference in TMS-induced interhemispheric signal propagation pre- and post-iTBS to the three brain regions. (**b**) Comparisons of the change of ISP among the three conditions. Error bars are standard diversions. ISP: interhemispheric signal propagation; M1: primary motor cortex; SMA: supplementary motor area; V1: primary visual cortex.

Although the grand-averaged TEP waveform showed that N100 arose in the central and right-lateral areas, N100 of three participants in one or more conditions was more prominent in the left than in the right hemisphere, reflecting individualized patterns of late TEP peaks [42]. Therefore, these ISPs were far higher than 100%, and were excluded from subsequent statistical analysis. A one-way rmANOVA test indicated that ISPs pre-iTBS were comparable between the three conditions (F = 0.28, *p* = 0.733). A two-way rmANOVA showed marginally significant effects of time (F = 3.38, *p* = 0.076) and interaction of time × condition (F = 2.70, *p* = 0.084), presented in Figure 4. Paired t-tests revealed that only iTBS to the M1 (*t* = 2.53, *p* = 0.030) reduced ISP, whereas neither iTBS to the SMA (*t* = 1.46, *p* = 0.175) nor to the V1 (*t* = −0.84, *p* = 0.423) significantly affected ISP. We found no significant difference between the decrements of areas under TEP curves of C3 and C4 (C3: −1.14 ± 1.01; C4 = −0.96 ± 2.09; *t* = −0.26, *p* = 0.798) in the M1 condition.

### 3.5. TMS-Induced Oscillations

**Figure 5:**
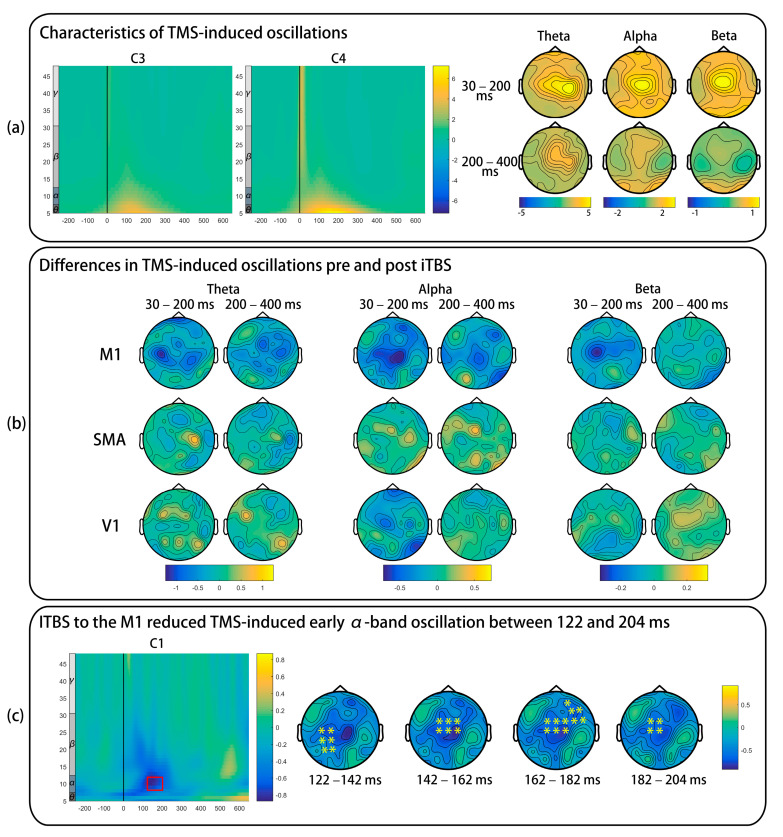
The effect of iTBS in TMS-induced oscillations. The power of each frequency was normalized by dividing all time bins by the mean of baseline power (−650 to −350 ms), and an absolute baseline correction was also carried out. (**a**) Only pre-iTBS data of the M1 condition are presented. (**b**) Topographical plots are the difference of early (30–200 ms) and late (200–400 ms) oscillations. (**c**) The red rectangle indicates significantly decreased TMS-induced early α-band oscillation. “*” represents electrodes with a significant difference. TMS: transcranial magnetic stimulation; iTBS: intermittent theta burst stimulation; M1: primary motor cortex; SMA: supplementary motor area; V1: primary visual cortex.

As presented in Figure 5a, TMS-induced oscillations in θ-, α-, and β-band increased in the early time window and gradually decreased in the late time window. Figure 5b shows the difference in TMS-induced oscillations pre- and post-iTBS. iTBS to the M1 significantly decreased TMS-induced early α-band synchronization, and this significant cluster started from 122 to 204 ms (*p* = 0.020) and distributed in the left-posterior, central, and right-anterior areas (Figure 5c). No significant differences were found in the remaining comparisons (all ps > 0.025).

### 3.6. Relationship between TMS-Induced Oscillations and the N100 Amplitude

**Figure 6:**
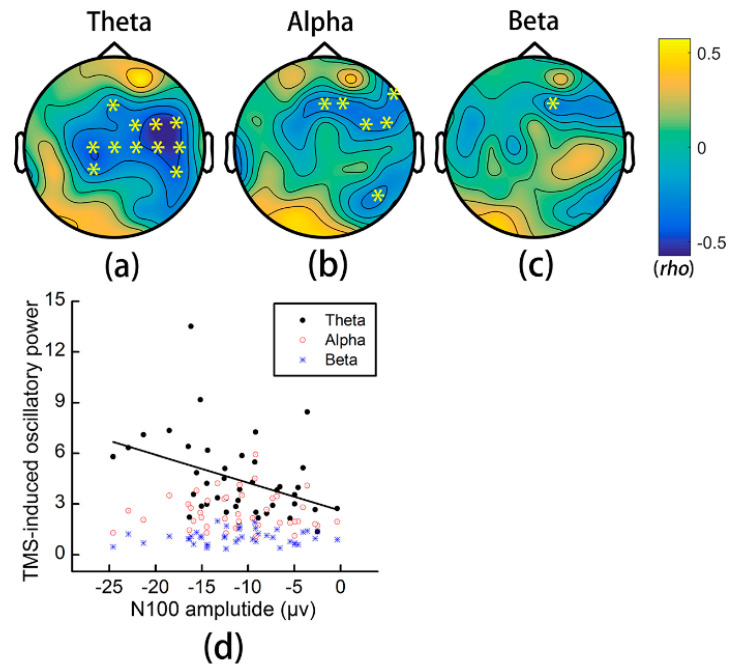
Relationship between the N100 amplitude of M1 TEPs and TMS-induced oscillations. (**a**–**c**) Correlation coefficients between the N100 amplitude of M1 TEPs and TMS-induced early θ-, α-, and β-band synchronization. “*” represents electrodes with significant correlation. (**d**) Scatter plots of the relationship between oscillations and the N100 amplitude of a representative electrode (Cz). TMS: transcranial magnetic stimulation.

The N100 amplitude of M1 TEPs had more robust significant correlation with TMS-induced θ-band oscillatory power in the central and right-lateral areas (highlighted in yellow, all ps < 0.05) than with α- or β-band oscillatory power (Figure 6).

## 4. Discussion

The current study investigated the effect of iTBS applied to three different brain areas in motor performance, TEPs, and TMS-induced oscillations. Our study demonstrated that iTBS to the M1 decreased the N100 amplitude of M1 TEPs and GMFP of the predefined time window of N100. Moreover, the decreased N100 amplitude was prominent in the contralateral hemisphere, accompanied by a decreased ISP from the stimulated hemisphere to the contralateral hemisphere sides. iTBS to the M1 also decreased TMS-induced early α-band oscillation. Although iTBS to the right SMA tended to induce faster motor execution, no significant changes were observed in the neurophysiological indices. As expected, iTBS to the V1 did not significantly change behavior performance, TEPs, or TMS-induced oscillations.

### 4.1. Effects of iTBS on TMS-Evoked Potentials

Concurrent TMS-EEG recording is extensively studied in probing cortical excitability of the dorsolateral prefrontal cortex and M1. The origin of TEPs is hypothesized to be the spatial and temporal summation of excitatory and inhibitory post-synaptic potentials of pyramidal neurons and interneurons following TMS pulses [9,43]. Specifically, the N100 amplitude of M1 TEPs correlates with GABAB-mediated inhibition [9,13]. Previous studies reported an increased N100 amplitude following iTBS to the dorsolateral prefrontal cortex [18,44]. However, we also noted inconsistent findings regarding the N100 amplitude of M1 TEPs before and after iTBS targeting the cerebello-thalamo-cortical pathway [19,45]. With regard to our findings, first, we observed no significant changes in early peaks of TEPs that were in line with the findings of another TMS-EEG study [20]. Although a moderate correlation between the change of N15-P30 amplitudes and the change of MEP amplitudes was found, suggesting potentially similar mechanisms underlying the two indices [20], the early components of TEPs, for example, P30, might not be sensitive in detecting enhanced cortical excitability, probably owing to its small size and potential contamination of TMS-induced noise and sensory-induced potentials. On the other hand, a recent study found that the antagonist of NMDA receptors did not significantly suppress P30 [46], but the early peak was decreased after intaking carbamazepine [47], a voltage-gated sodium channel blocker. These two studies indicated that the early peak of TEPs (P25 or P30) produced by a fixed intensity based on RMT cannot reflect the full picture of cortical excitability and may miss some specific information relevant to the function of excitatory receptors.

The most significant finding in the current study was that iTBS to the M1, but not the SMA and V1, reduced the N100 amplitude of M1 TEPs and GMFP. As the N100 amplitude correlates with the GABAB-mediated cortical inhibition, our study implies that iTBS to the M1 might downregulate the function of GABAB-mediated inhibitory circuits within the M1 [9]. Epidural recording after iTBS demonstrated enhanced later I-waves with unchanged I1-wave, suggesting substantial modulatory effects prior to the pyramidal tract neurons [48], probably taking place in the GABAergic interneurons. Our finding is also supported by previous studies in which the level of GABA neurotransmitters reduced after iTBS [49] and increased after cTBS [50]. In our study, we did not find any significant alteration in the amplitude of N45 or P55, which may be mediated by GABAA receptors. Previous studies also showed inconsistent findings regarding the effect of TMS on these peaks [17,18,20,45,51], and more studies are needed to clarify these findings.

LICI and cortical silent period are used to probe GABAB-mediated cortical inhibition. Evidence from TMS-EEG studies revealed that the N100 amplitude of M1 TEPs was moderately correlated with LICI [52] and the duration of the cortical silent period [53]. Previously, iTBS with a subthreshold intensity was found to be ineffective in changing LICI [54] and the cortical silent period [55]. However, 1 Hz repetitive TMS with suprathreshold intensity could significantly lengthen the cortical silent period [55] and increase the N100 amplitude [17]. Besides, LICI was enhanced along with the increase of conditioning intensity [56], but reversed to facilitation by a subthreshold conditioning pulse [57]. Therefore, GABAB-mediated intracortical inhibition may be more easily evoked and modulated by suprathreshold stimuli than by subthreshold stimuli. As in the aforementioned studies, theta burst stimulation employs subthreshold stimuli that may not dramatically affect the function of GABAB-mediated inhibitory circuits, resulting in no changes in LICI [54]. In contrast, our study demonstrated the capability of TEPs in detecting decreased function of GABAB-mediated inhibition reflected by the decreased N100 amplitude.

Our study also found that only iTBS to the M1 decreased ISP. A few previous studies investigated the effect of TMS on ISP. Baclofen was shown to increase interhemispheric inhibition [58], and it was recently shown to decrease ISP in the M1 [15], establishing a potential link between ISP and interhemispheric inhibition. In addition, Premoli et al. [13] showed that baclofen increased N100 in the stimulated hemisphere only, instead of in bilateral sides. Therefore, baclofen is expected to decrease ISP because of the increased N100 amplitude in the stimulated hemisphere [15] and no significant changes in the contralateral hemisphere. According to these pharmacological studies, increased N100 and decreased ISP might be interpreted as upregulated function of GABAB-mediated intracortical/interhemispheric inhibition. However, we found that iTBS decreased ISP and N100 in bilateral hemispheres, which seemed not to be compatible with each other. To explain the issue, we reviewed the comparison on TEPs and the decrements of areas under TEP curves of C3 and C4. We can easily note that the electrodes with significant changes distribute in bilateral areas and are even more robust in the contralateral than in the stimulated hemispheres (Figure 2c). Furthermore, the decrements of areas under TEP curves were not significantly different between C3 and C4, also supported by Chung et al. [18] in which the effect on N100 was almost symmetrically distributed in both hemispheres after iTBS to the dorsolateral prefrontal cortex. Therefore, we think that the decreased ISP found in our study reflects that iTBS might not only locally reduce the intracortical inhibition within the stimulated hemisphere, but also show disinhibition in the contralateral hemisphere. Our notion is also supported by previous studies showing remote effects of iTBS [54,59]. In addition, Premoli et al. [13] found that positive modulators of GABAA receptors, such as alprazolam, decreased the N100 amplitude in the contralateral hemisphere and had unchanged N100 in the stimulated hemisphere. Therefore, these positive modulators of GABAA receptors are also expected to decrease ISP. Although both GABAA and GABAB drugs may decrease ISP, we think the interhemispheric dynamics induced by the two types of drugs should be distinctly different, and the neurophysiological significance of ISP needs more investigation.

The behavior test indicated that iTBS to the SMA tended to shorten RT of the left hand, which was in line with previous studies showing a role of the SMA in facilitating sequential motor performance [27]. However, no significant differences were found in TEPs and TMS-induced oscillations in any frequency band or electrode. The SMA has reciprocal connection with the M1, and previous studies have already demonstrated the facilitatory effect of the SMA to the M1 [6,7,60,61]. However, excitatory stimulation over the SMA could not decrease the SICI or LICI within the M1, suggesting that the facilitatory effects from the SMA to the M1 are not caused by local disinhibition [61]. In addition, we did not find a significant change on P30, which was initially expected to increase. Moreover, we also would like to point out that structural images-guided navigation was not used in our study; therefore, the precision of our method for localizing the SMA might be questioned. Overall, our TMS-EEG study supported previous findings that increased excitability of the M1 by excitatory stimulation to the SMA is not likely caused by disinhibition within the M1.

### 4.2. Effects of iTBS on TMS-Induced Oscillations

We analyzed TMS-induced oscillations characterized by early α- and β-band synchronization and late β-band desynchronization [39]. As shown in Figure 5, θ-band oscillatory power highly topographically overlapped with the distribution of the N100 amplitude, but α- and β-band oscillations did not. The most prominent finding was the decreased early synchronization in α band after iTBS to the M1, distributed in left-posterior, central, and right-anterior areas. A recent pharmacological study found that the early α-band synchronization can be increased and decreased by GABAA and GABAB drugs, respectively [39]. Because we had observed decreased N100 amplitudes after iTBS, which indicated downregulated GABAB-mediated inhibition, we can rule out that the decreased early α-band synchronization was caused by upregulating GABAB-mediated inhibition. In contrast, the decreased early α-band synchronization might indicate the downregulation of GABAA inhibition. However, previous studies showed inconsistent findings regarding the effect of theta burst stimulation on SICI [54,55] and we also did not find changes in N45 or P55. Therefore, we could not come to a firm conclusion on the effect of iTBS in GABAA-mediated inhibition based on current findings.

Although the N100 amplitude of M1 TEPs significantly decreased after iTBS, no significant changes were found in early β- or late α-band desynchronization. The possible reasons were twofold: first, we did not find a significant correlation between early β-band oscillatory power and the N100 amplitude, which may explain the absence of significant alteration in early β-band synchronization; second, somatosensory re-afferent caused by twisted fingers may mask the effect in late oscillations [39]. Several studies have investigated TMS-evoked oscillations after theta burst stimulation [18,45,51], but they did not analyze TMS-induced oscillations. It was reported that TMS-evoked α-band synchronization of the M1 decreased by cTBS to the cerebellum [45], while θ- and γ-band TMS-evoked and total (i.e., evoked + induced) oscillations increased after iTBS to the dorsolateral prefrontal cortex [18]. TMS-evoked oscillatory power can measure the “phase-reset” effect of TMS pulses for ongoing oscillations, whereas TMS-induced oscillatory power may reflect subsequent endogenous oscillations [62]. Taken together, available studies revealed that TMS-evoked and -induced oscillations are potential markers of changed cortical excitability. However, the current findings are still preliminary, and more studies are needed to explain the significance of TMS-evoked and -induced oscillations, particularly in patients with various neurological and psychiatric conditions.

Our study has limitations. First, appropriate TMS measures based on MEPs were not administrated in our study, resulting in inconclusive findings compared with previous studies in which the after-effects of iTBS were investigated. Second, intensity for TMS-EEG recording was set at 110% of RMT, which could induce considerable muscle twitching over the contralateral hand; thus, sensory-evoked potentials were produced and contaminated with late components of TEPs, such as N100 and P180. Third, we excluded three participants from the analysis on ISP because of their far higher values than 100%. We do not have a clear explanation for why they had more prominent N100 in the left than in the right hemisphere, and future study may investigate how ISP is associated with individualized brain networks.

## 5. Conclusions

Our study showed that iTBS to the SMA and V1 did not significantly alter TEPs and TMS-induced oscillations. However, iTBS to the M1 reduced the N100 amplitude of M1 TEPs in bilateral hemispheres, suggesting its local and remote after-effects in modulating cortical excitability. Further exploration of TMS-induced oscillations after iTBS for changed cortical excitability in patients with various neurological and psychiatric conditions is warranted.

## Data Availability

The data presented in this study are available on request from the corresponding author.
